# Heuristics for Multiobjective Optimization of Two-Sided Assembly Line Systems

**DOI:** 10.1155/2014/458959

**Published:** 2014-03-20

**Authors:** N. Jawahar, S. G. Ponnambalam, K. Sivakumar, V. Thangadurai

**Affiliations:** ^1^Department of Mechanical Engineering, Thiagarajar College of Engineering, Madurai, Tamilnadu 625 015, India; ^2^Advanced Engineering Platform and School of Engineering, Monash University Malaysia, 46150 Bandar Sunway, Malaysia; ^3^Department of Manufacturing Engineering, Central Institute of Plastics Engineering & Technology, (Government of India), Chennai, Tamilnadu 600 032, India

## Abstract

Products such as cars, trucks, and heavy machinery are assembled by two-sided assembly line. Assembly line balancing has significant impacts on the performance and productivity of flow line manufacturing systems and is an active research area for several decades. This paper addresses the line balancing problem of a two-sided assembly line in which the tasks are to be assigned at L side or R side or any one side (addressed as E). Two objectives, minimum number of workstations and minimum unbalance time among workstations, have been considered for balancing the assembly line. There are two approaches to solve multiobjective optimization problem: first approach combines all the objectives into a single composite function or moves all but one objective to the constraint set; second approach determines the Pareto optimal solution set. This paper proposes two heuristics to evolve optimal Pareto front for the TALBP under consideration: Enumerative Heuristic Algorithm (EHA) to handle problems of small and medium size and Simulated Annealing Algorithm (SAA) for large-sized problems. The proposed approaches are illustrated with example problems and their performances are compared with a set of test problems.

## 1. Introduction

An assembly line is a manufacturing process in which components are consecutively assembled to an unfinished product depending on a set of tasks to produce a final product. The unfinished product is moved from one station to its successive station until they reach to the end of the line. In an assembly line, each task which is performed in a certain time called as the task time is allocated to stations according to a given precedence relationship among tasks. Each and every station is assigned with a set of different tasks. At each station, the set of task allotted to it is performed in a limited time called as the cycle time. The assembly line balancing problem (ALBP) is to assign the tasks to the work centers while optimizing one or more objectives without violating restrictions imposed on the line. Assembly lines falls under two categories: one-sided assembly lines and two-sided assembly lines. In a one-sided assembly line [1] only one side (either left side “L" or right side “R") of the line is used, whereas both L and R of the line are simultaneously used in a two-sided assembly line [2]. The main difference between one-sided and two-sided lines is the constraints in the assignment of tasks. The one-sided line needs to satisfy the precedence relationship among the tasks. Whereas, in two-sided assembly line the certain tasks are constrained to a specific side (i.e., L or R) in addition to the precedence constraints. According to Bartholdi [3], in practice a two-sided line can provide several advantages over a one-sided line, like the reduction of (i) the number of operators, (ii) the throughput time, (iii) the cost of tools and fixtures, as they can be shared by the operators of both sides, (iv) material handling costs, and (v) length of the assembly line. This paper addresses the line balancing problem of a two-sided assembly line in which the tasks are to be assigned at L side or R side or any one side (addressed as E).

Assembly line balancing has significant impacts on the performance and productivity of flow line manufacturing systems and is an active research area for several decades. The two general objectives of line balancing are minimisation of number of workstations to meet the production rate [4–6] and maximisation of production rate with given number of work stations [7]. The other objectives are minimisation of the cycle time for a given number of mated station ([2, 8, 15]); minimisation of number of mated stations for a given cycle time, that is, line length and number of positions [9, 10]; minimisation of number of tasks assigned to each workstation [8]; maximisation of work relatedness and slackness [1, 6]; assigning of tasks from left station to right station of the position based on the start time of the tasks [11]. Minimum number of workstations is the widely used objective since it provides advantages in terms of less workspace or line length, minimum workforce, a smaller amount of material movement of tools and materials. When the tasks are not well balanced among the stations it may lead to excess work to some stages, and consequently idleness to some stations. This necessitates that assembly line work elements are to be well balanced among workstations for single piece flow. On this consideration, this paper considers minimum number of workstations and minimum unbalance time or minimum of maximum idle time as the optimality criteria to the two-sided assembly line balancing problem (TALBP).

The approaches to solve multiobjective optimization problem are broadly divided into two categories [12]. The first approach is to combine all the objectives into a single composite function or to move all but one objective to the constraint set. The second approach is to determine the Pareto optimal solution set, solutions that are nondominated with respect to each other. Pareto optimal solutions are often preferred to single solutions because they can be practical when considering real-life problems since the final solution of the decision maker is always a tradeoff. Unlike the first approach, Pareto optimization tool provides a solution set in which solutions are nondominated with respect to each other. This tool is chosen for the proposed problem, because the problem is of a minimization type with multiple objectives. The Pareto optimal method will give the set of solutions from which the user can choose depending upon the requirement. On the above concern, this paper attempts to evolve Pareto optimal front for the two objectives of minimum number of workstations and minimum unbalance time to the TALBP.

The TALBP belongs to NP-hard class of combinatorial optimization problems [3]. Besides, the problem under consideration considers two objectives that add complexity to the problem. The combinatorial structure and complexity of the bicriteria optimisation to TALBP make it difficult to obtain an optimal solution. Further complexity increases further with increase in problem size. A large number of methods for solving one-sided assembly line balancing problem have been studied, including heuristic procedures and exact algorithms. Recently, some heuristic algorithms have been proposed to solve TALBP. Kim et al. presented an approach based on genetic algorithm to provide solution for TALBP [2]. Fleszar and Hindi [13] proposed an enumerative heuristic and reduction method for the assembly line balancing problem. Kim et al. [2] used genetic algorithm to solve two-sided assembly line balancing to minimize the number of workstations to which tasks are allocated. Lee et al. [1] proposed a group assignment procedure focusing on the maximization of work relatedness and work slackness with a little or no loss in cycle time and the number of stations. Hu et al. [11] proposed a station-oriented enumeration algorithm that is integrated with the Hoffmann heuristic (2003). Baykasoglu and Dereli [6] used ant-colony based heuristic for two-sided assembly line balancing problem to minimize the number of workstations and maximize the work relatedness. Kim et al. [2] used genetic algorithm to solve two-sided assembly line balancing. Simaria and Vilarinho [4] implement ant-colony optimization for mixed two-sided assembly line balancing. On the other hand, Özcan and Toklu [8] developed a basic mathematical model for the TALBP. Rubiano-Ovalle and Arroyo-Almanza [14] proposed a Memetic Algorithm for solving deterministic two-sided assembly line balancing problem. Özcan [9] used mixed integer programming and simulated annealing for stochastic two-sided assembly line balancing. Xiaofeng et al. [10] proposed a branch and bound algorithm to solve two-sided assembly lines problems. Purnomo et al. [15] used genetic algorithm and iterative first fit rules to solve the TALBP with assignment restrictions. The above review reveals that heuristics and metaheuristics have been used extensively to solve TALBP. On the similar thoughts, this paper proposes two heuristics to evolve optimal Pareto front for the TALBP under consideration and are as follows: Enumerative Heuristic Algorithm (EHA) to handle problems of small and medium size and Simulated Annealing Algorithm (SAA) for large-sized problems.

The reminder of this paper is organized as follows.  Section 2 describes the multiobjective TALBP under consideration. Sections 3 and 4 delineate the proposed EHA and SAA along with illustrations.  Section 5 discusses the performance of the proposed algorithms by comparing their solutions with standard problems taken from the literature.  Section 6 presents the summary of the research along with future research directions.

## 2. Problem Description 

**Figure 1:**
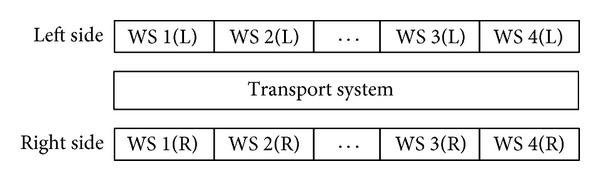
Configuration of two-sided assembly lines [4].

The problem under consideration is a two-sided assembly line [9] in which products such as car, trucks, and heavy machineries that are larger in size and shape are manufactured involving L, R, and E tasks.  Figure 1 shows the arrangement of two-sided assembly lines. The line has two sides, left and right, and, in most cases, at each position there is a pair of workstations directly facing each other. The two opposite operators perform, in parallel, different tasks on the same individual item.

The line is assumed as inline assembly and the workers/automatic processing heads are arranged on both sides of the line. The number of tasks involved in the assembly of them depends on the product structure and in the general sense is taken as “*N*.” Each task “*i*” is constrained with certain predecessor tasks. The precedence relationships among tasks are known. In addition to the precedence constraints, some of the tasks are restricted to any one side (L or R) of the assembly line and other remaining tasks can be assigned to either side (E) of the line. The time “*t*_*i*_” for processing task “*i*” is known for all tasks and deterministic. The cycle time “CT” is fixed based on the production target and is known. Besides, the following assumptions are made: operators perform their tasks in parallel at both sides of the line simultaneously within a given fixed cycle time and the move times of operators are included in task times. The objective of minimization of unbalance time among workstations is considered additionally with the general objective of minimization of number of workstations for the specified cycle time “CT” (or the production target). The problem can be stated as determination of optimal assignment of tasks to workstation for minimum unbalance and number of work stations target given the followings: cycle time “CT,” number of tasks “*N*,” precedence and side constraints (L or R or E) for each task, and processing time (*t*_*i*_) for all tasks (*i* = 1 to *N*).

## 3. Enumerative Heuristic Algorithm (EHA)

**Figure 2:**
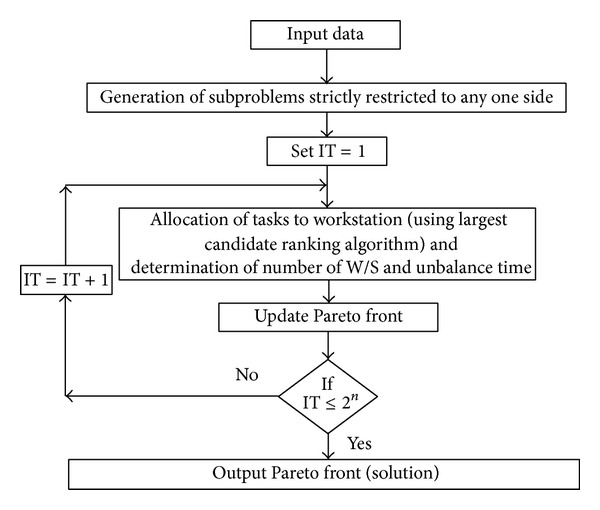
Frame work of the EHA.

Figure 2 shows the framework of the proposed EHA. The various modules are described in this section.

*Data Input*. The task related data to the TALBP under consideration are given as input in this module and are as follows:

- number of tasks “*N*,”
- processing time for task “*t*_*i*_” for all tasks (∀_*i*_, *i* = 1 to *N*),
- operation direction of task “*k*_*i*_” for all tasks (∀_*i*_, *i* = 1 to *N*), where L(1), R(2), and E(3),
- number of precedence tasks “nop_*i*_” for all tasks (∀_*i*_, *i* = 1 to *N*),
- set of immediate precedence tasks “*p*_*i*_” for all tasks (∀_*i*_, *i* = 1 to *N*),
- cycle time “CT,” where CT ≥ Max *t*_*i*_ and CT ≤ ∑_*i*=1_^*N*^ *t*_*i*_.

**Figure 3:**
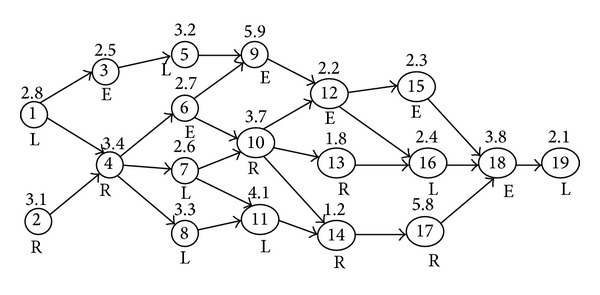
Precedence diagram along with task time and operation directions of P19 - TALBP.

Consider a sample TALBP (used to illustrate the EHA and addressed hereafter as “P19") that involves 19 tasks in which 7 and 6 tasks are restricted to left and right sides, respectively, and the remaining 6 tasks can be performed on either side.  Figure 3 shows the precedence and side restrictions of the tasks along with their work element times. The number given inside, above and below the nodes, indicates the task number “*i*,” the processing time “*t*_*i*_,” and the operation directions “*k*_*i*_”. The arrows indicate the precedence relationship for each task. The task related data given in Table 1 that corresponds to Figure 3 and the CT, which is assumed as 6 minutes, are given as input to EHA.

*Generation of Subproblems (“E” Type Tasks Restricted to Either L or R Side)*. The tasks that belong to “E” category can be and are to be done either “R” on “L.” This module generates all possible assignments of “E” type tasks into L or R type tasks and provides a number of subproblems of two-sided assembly line, in which all the tasks are strictly restricted to either one side (i.e., L or R). The number of subproblems depends on the number of “E” type tasks (*n*) and is equal to 2^*n*^. The number of “E” type tasks to the P19 problem is 6.  Table 2 shows the 64 different possible assignments of the six “E” type tasks to the P19 problem considered for illustration. This provides 64 subproblems with tasks that are strictly restricted to either one side (i.e., L or R).

*Initialization of Enumeration Counter*. All possible assignments need to be evaluated for the problem objectives. In order to explore all of them, an iteration counter “IT" is used and is set equal 1 in this module.

*Allocation of Tasks to Workstations and Evaluation*. Considering one subproblem at a time, this module allocates the tasks to workstations arranged at both sides work stations, “W(L) and W(R)," by applying the logic of the Largest Candidate Ranking Algorithm [16] modified suitably for subproblems of TALBP. The allocation follows a five-step procedure as given below.

**Step 1**

Arrange the tasks in the descending order according to their precedence relations.

**Step 2**

Select the task with the largest task time from the tasks that have already satisfied precedence constraint.

**Step 3**

Assign the selected task to the L or R side workstation according to the direction restrictions provided balance time is available in the workstation. New workstation is added when the available balance time in the workstation is less than the task time of the selected task.

**Step 4**

Repeat Steps 2 and 3 till all the tasks are allotted to workstations.

**Step 5**

Determine the objective criteria of number of workstations and maximum unbalance time based on the allocations made to the “E” type assignments.

Table 3 shows the allocation of tasks corresponding to subproblem 1 (i.e., 1st possible assignment) of P19. The number of workstations and maximum unbalance time to subproblem 1 of P19, respectively, are 14 (left-side workstations = 9; right-side workstation = 5) and 4.8 min.

*Updation of Pareto Front*. This step updates the Pareto solutions based on the principle of dominance by comparing the objective functions values of the current assignment and the objective function values of the existing Pareto solutions set. As no Pareto solution set is available during the 1st iteration and the assignment of first iteration is added to the Pareto solution set. At the beginning of EHA, the solution corresponds to assignment 1 of the sample problem P19 (i.e., Number of W/S—14 and maximum unbalance Time—4.8 min) thus becomes the Pareto solution set after the first iteration. In the 2nd iteration, the subproblem 2 is solved and Table 4 shows the results of it. The solution corresponding to assignment 2 of P19 is Number of W/S—14 and maximum unbalance Time—4.7 min. Hence, the 2nd assignment solution dominates the 1st assignment solution and the Pareto solution set gets updated with the 2nd solution and 1st assignment exits from the Pareto set. The 3rd assignment results in 13 workstations with 4.8 as the maximum unbalance time. Its solution given in Table 5, when compared with the existing Pareto solutions, is superior with respect to number of workstations and inferior in terms of unbalance time and thus the Pareto solution set is appended with this solution. The solutions of 2nd and 3rd assignments thus becomes the updated Pareto solution set. The process of updating Pareto solution set continues till all assignments are evaluated.

**Figure 4:**
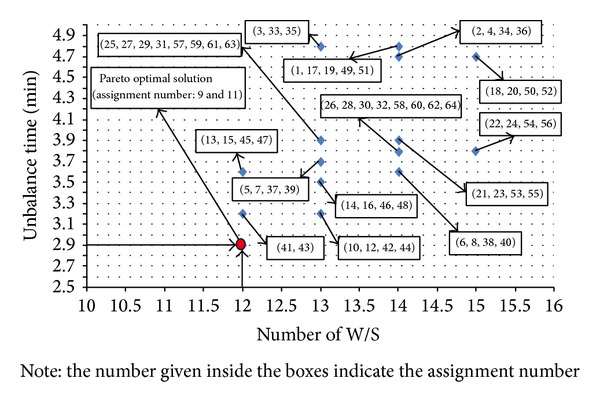
Pareto front for problem P19 of proposed EHA.

*Termination Check and Output*. This step checks whether all possible assignments are evaluated. When the iteration counter exceeds 2^*n*^ (i.e., the possible number of assignments), the updating of Pareto front stops and proceeds to provide the output. Otherwise, the iteration counter is incremented by one and goes to allocation and evaluation module.  Figure 4 shows the solution output. The nondominated solution set with respect to minimum number of workstations (12) and minimum maximum unbalance time (2.9 min) becomes the Pareto solution set. Assignments corresponding to 9 and 11 provide optimal Pareto solution set after 64 iterations. Tables 6 and 7 provide the solutions to 9th and 11th assignments, which are optimal solutions to the problem P19.

## 4. Simulated Annealing Algorithm

**Figure 5:**
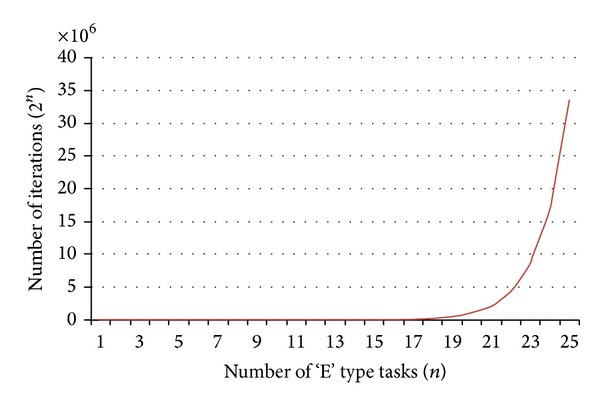
Number of iteration of EHA Vs number of “E” type tasks.

The EHA, an iterative procedure, requires large computational effort for large size problems. The number of iterations to be performed (i.e., 2^*n*^) increases exponentially with the number of “E” type tasks “*n*.” Figure 5 indicates the required number of iterations for different values of “*n*.” This restricts its application to large size problems. Meta heuristics, such as genetic algorithm (GA), ant colony optimization (ACO), Particle Swarm Optimization (PSO), Tabu Search (TS), and Simulated Annealing Algorithm (SAA) have the capability of searching intelligently in larger solution space and can provide solution quicker than the iterative procedure. SAA is commonly said to be the oldest among the metaheuristics and surely one of the first algorithms that had an explicit strategy to avoid local minima.

The SAA is derived from the field of statistical mechanics. It follows a slow cooling process called “annealing” to estimate the ground state energy of a matter [17]. Metropolis and his colleagues developed an algorithm based on annealing principle to simulate a solid to thermal equilibrium. Kirkpatrick et al. [18] successfully illustrated the application of this algorithm to optimize a combinatorial problem. The fundamental idea is to allow moves resulting in solutions of worse quality than the current solution (uphill moves) in order to escape from local minima. The acceptance of deteriorated solution is probabilistically determined by the Metropolis Criterion (*P*) as given by

(1) $$\begin{array}{c} P\;=e^{\left(-(X_{p}-X)/T\right)}, \end{array}$$

where *X* is the solution at current state, *X*_*p*_ is the perturbated solution of the system at new state, and *T* is the control parameter (temperature). The algorithm begins with an initial solution (randomly generated) and a high temperature. The second solution is accepted directly, provided it has a smaller functional value (fitness) than the first solution; otherwise, it is accepted with a probability, which is obtained from (1). This completes an iteration of the SAA procedure. In the next generation, using the perturbation scheme, the neighborhood of the current solution creates another solution and checks for acceptance or rejection. In order to simulate the thermal equilibrium at every temperature, a number of solutions are tested at a particular temperature before reducing the temperature. The algorithm is terminated when a sufficiently small temperature is obtained or a small enough change in the objective function value is found. Simulated annealing performs better than any local optimization method and yields a solution close to global optimum [19]. It is mainly attributed to the occasional acceptance of the worse solution, which enables to escape from being trapped at the local minimum. On these considerations, this paper proposes SAA to handle larger problems.

### 4.1. Framework of SAA

**Figure 6:**
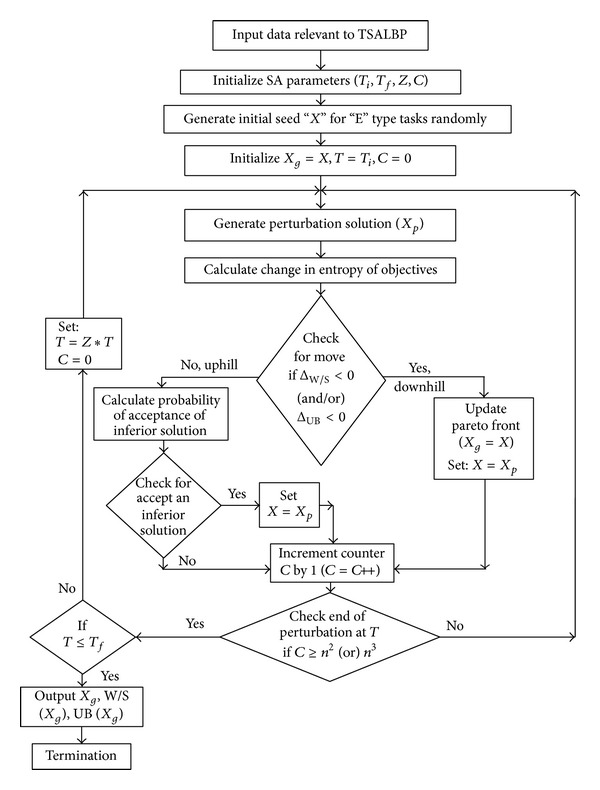
Framework of the proposed Simulated Annealing Algorithm for TSALBP.

Figure 6 provides the framework of the proposed SAA. This section delineates the details of the various steps of the SAA that is proposed to evolve the pareto front for the objectives of minimum number of workstations and minimum of maximum unbalance time among workstations.

### 4.2. Procedural Steps of the Proposed SAA

#### 4.2.1. Input

**Figure 7:**
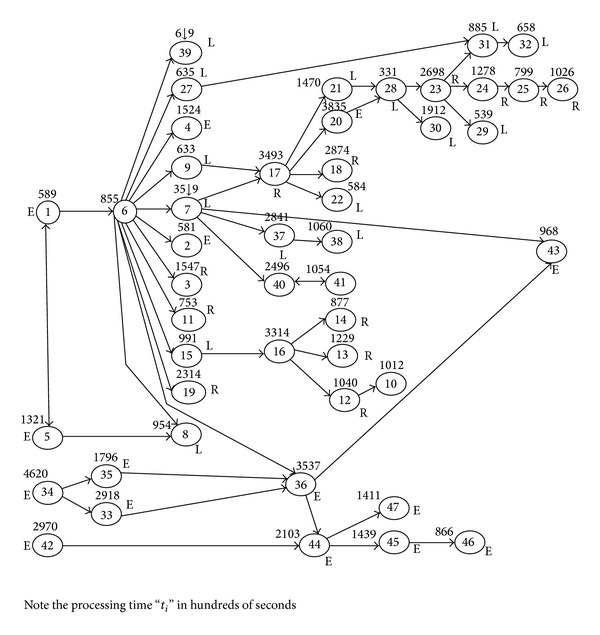
Precedence diagram along with task time and operation directions of 47 tasks TSALBP (Source: [14]).

The data relevant to the problem are given as input to SAA.  Figure 7 provides the TALBP used for illustration (Source: [14]) of proposed SAA.  Table 8 provides the input data for the illustration problem P47 in which the number of “E” type tasks is 25. The number given inside and above and left, right, and below the nodes indicates the task number “*i*,” the processing time “*t*_*i*_,” and the operation directions “*k*_*i*_.” The arrows indicate the precedence relationship for each task.

#### 4.2.2. Initialization of SAA Parameters and Counters

The parameters that influence the performance of SAA are initial temperate *T*_*i*_, temperature reduction factor “*Z*,” number of perturbations at each temperature “*C*,” and final temperature *T*_*f*_. The parameter temperature of the algorithm decides the probability of acceptance of the inferior solutions. The probability of acceptance at the beginning of the algorithm is normally set around 0.9. This is used to set the value of “*T*_*i*_.” The other parameters “*Z*”, “*T*_*f*_,” and “*C*” decide the exploration requirements of SAA which primarily depend on problem size/solution space [20]. Based on trials, the parameters are set as follows: *T*_*i*_ = 450°C; *T*_*f*_ = 20°C; *Z* = 0.95; *C* = *n* ^3^ for large size problems (or) *n* ^2^ for small size problems.

#### 4.2.3. Generation of Current Seed

Each “E” type task is assigned to either left side (coded as 1) or right side (coded as 2) by random process. In a string of length “*n*,” the choice of assignment (1 or 2) of a bit at position “*j*” corresponds to the *j*th “E” type tasks from the list of “E” tasks arranged in ascending order of their node numbers. This string becomes the initial seed “*X*” that represents one assignment of E type tasks to L or R.  Table 9 shows a current seed “*X*” that is generated randomly for the illustration problem “P47.”

#### 4.2.4. Initialization of Pareto Front (*X*_*g*_), Temperature (*T*), and Perturbation Counter (*C*)

The following values are set as initial values to pareto front (*X*_*g*_), current temperature of SAA (*T*), and counter (*C*): *X*_*g*_ = *X*(*j*, *i*, *k*_*i*_), *T* = *T*_*i*_ (450°), *C* = 0;

#### 4.2.5. Generation of Perturbation Seed (*X*_*p*_)

The initial seed is perturbed randomly to yield another solution which is called a perturbed solution, “*X*_*p*_.” In order to avoid redundancy, the perturbation mechanism is made purely random. A perturbed string (*X*_*p*_) is generated in the following manner. Four random numbers are generated between 1 to *n*. The choice of assignments (1 or 2) in those four positions of “*X*” is changed to the opposite choice (i.e., if the choice in *X* is 1, it is changed to 2 and vice versa).  Table 10 shows the perturbation seed “*X*_*p*_” to the initial seed given in Table 9, which is generated with the random numbers (*r*), generated 4, 11, 16, and 19.

#### 4.2.6. Calculation of Change in Entropies of Objective Criteria

The objective functions of number of workstations and unbalance time are found for the two assignments represented in “*X*” and “*X*_*p*_” using the steps described in allocation of tasks to workstations and evaluation module of Section 3. Let W/S(*X*) and W/S(*X*_*p*_) be the number of workstations corresponding to the assignments given in *X* and *X*_*g*_, respectively, and let UB(*X*) and UB(*X*_*p*_) be the unbalance time corresponding to the assignment given in *X* and *X*_*g*_, respectively. Then change in entropies for the workstation Δ*E*_W/S_ and the unbalance time Δ*E*_UB_ are calculated using (2) and (5), respectively:

(2) $$\begin{array}{c} \Delta E_{\text{W}/\text{S}}=\text{W}/\text{S}\left(X_{p}\right)-\text{W}/\text{S}\left(X\right), \end{array}$$

(3) $$\begin{array}{c} \Delta E_{\text{UB}}=\text{UB}\left(X_{p}\right)-UB\left(X\right). \end{array}$$

The change in entropies is given below:

(4) $$\begin{array}{c} \Delta E_{\text{W}/\text{S}\;}=\text{W}/\text{S}\left(X_{p}\right)-\text{W}/\text{S}\left(X\right)=9-9=0,\\ \Delta E_{\text{UB}}=\text{UB}\left(X_{p}\right)-\text{UB}\left(X\right)=64.48-78.34=-\text{ve}. \end{array}$$

#### 4.2.7. Check for Move

This step directs the further steps of SAA to uphill move or downhill move to update initial seed (*X*) and Pareto front (*X*_*g*_). The algorithm takes the route of downhill move when Δ*E*_W/S_ < 0 or Δ*E*_UB_ < 0. Otherwise it proceeds with uphill move.

**Example 1**

When *X*_*p*_ = [1 1 1 2 1 2 2 1 2 1 2 1 1 1 2 1 2 1 1 1] that has WS(*X*_*p*_) = 9 and UB(*X*_*p*_) = 64.48 sec and *X* = [1 1 1 1 1 1 2 2 2 1 1 1 1 1 2 2 2 2 2 1] that has WS(*X*) = 9 and UB(*X*) = 78.34 sec, then the condition of Δ*E*_UB_ ≤ 0 is satisfied and results to downhill move. Suppose *X* and *X*_*p*_ are vice versa (say *X*′ = *X*_*p*_ and *X*_*p*_′ = *X*); then the condition is not satisfied and results to uphill move.

#### 4.2.8. Downhill Move

This module modifies the current seed (*X*) and updates the Pareto front (*X*_*g*_). First, Pareto front is updated by comparing the *X*_*p*_ and *X*_*g*_ using the procedure outlined in Section 3. Then the perturbed seed *X*_*p*_ is set as current *X*.

**Example 2**

When *X* = [1 1 1 1 1 1 2 2 2 1 1 1 1 1 2 2 2 2 2 1] and *X*_*p*_ = [1 1 1 2 1 2 2 1 2 1 2 1 1 1 2 1 2 1 1 1], then *X*_*g*_ = *X*_*p*_ and *X* = *X*_*p*_. That is, *X*_*g*_ = [1 1 1 2 1 2 2 1 2 1 2 1 1 1 2 1 2 1 1 1] and *X* = [1 1 1 2 1 2 2 1 2 1 2 1 1 1 2 1 2 1 1 1].

#### 4.2.9. Uphill Move

In this module, the perturbed seed (*X*_*p*_), though inferior to current seed (*X*) in both objectives, it is accepted with probability as current seed *X* allowing the algorithm to search for good solution in the other solution region. The steps involved in this process are as follows.

**Step 1**

Calculate the probability of accepting “*P*_*a*_" for the inferior “*X*_*p*_” using the formula given in

(5) $$\begin{array}{c} P_{a}=\frac{e^{-\;\Delta E_{\text{W}/\text{S}}/T}+e^{-\;\Delta E_{\text{UB}}/T}}{2}. \end{array}$$

**Step 2**

Generate random number “*r*” (0 to 1).

**Step 3**

If *r* ≤ *P*_*a*_, then modify *X* = *X*_*p*_; otherwise *X* = *X*.

**Example 3**

When *X*′ = [1 1 2 2 1 2 2 1 2 1 2 2 1 2 2 1 1 1 1 1] that has WS(*X*′) = 8 and UB(*X*′) = 16.74 sec and *X*_*p*_′ = [1 1 1 2 1 2 2 1 2 1 2 1 1 1 2 1 2 1 1 1] that has WS(*X*_*p*_′) = 9 and UB(*X*_*p*_′) = 64.48 sec, then the condition of Δ*E*_W/S_ ≤ 0 or Δ*E*_UB_ ≤ 0 is satisfied and proceeds with the uphill move.

#### 4.2.10. Check for Number of Perturbations

This step limits the number of perturbations performed at any temperature “*T*” to *n* ^3^ times for small size problems (or) *n* ^2^ times for large size problems. It is done giving an increment to the perturbation counter “*C*” and checking whether it is within the maximum number of perturbations set as *n* ^3^ small size problems (or) *n* ^2^ for large size problems. *C* is reset with the value of zero whenever a fresh *T* is set.

#### 4.2.11. Check for Termination

The perturbations and updates of Pareto front are carried out by reducing the *T* by *Z*∗*T* till it *T* reaches *T*_*f*_ (=20°C).

#### 4.2.12. Output Pareto Front

The updated Pareto front (*X*_*g*_) and their corresponding assignments are the output of the SAA. The final Pareto front to P47 is a single solution, the output of which is given below:

 
*X*_*g*_ = [1 1 2 1 1 2 1 2 2 1 2 1 2 1 2 1 1 2 2 1],
 W/S  [*X*_*g*_] = 8,
 UB  [*X*_*g*_] = 14.69 seconds.

## 5. Results and Discussions

Five data sets, illustrative problems P19 and P47 [14], and three more problems taken from the literature [2] and addressed as P9, P12, and P24 are used to study the performance of the proposed algorithms. Tables 12, 13, and 14 provide the data of P9, P12, and P24, respectively.  Table 11 shows the results obtained for the test data with EHA and SAA along with results reported in the source papers.

The comparison of results reveals the following.

- The solutions of EHA (except P47) and SAA match with the optimal solutions of the source papers to the objective of minimum number of workstations, besides meeting the other objective of minimum unbalance time among the work stations. This proves the capability of the proposed algorithms in handling multiobjective optimization.
- The EHA could not solve P47 problem due to the limitation in the array size of the program code and memory requirement for large computation.
- The repeatability of Pareto solution on 5 trails with SAA with *n* ^3^ as perturbation termination, when compared to *n* ^2^ as perturbation termination, especially with larger problems (P24 and P47), is higher. Though 100% repeatability could not be assured even with *n* ^3^ as perturbation termination, it has the potential to capture the near optimal solution. Further tuning the parameters of SAA can guarantee optimal solution in all runs.
- All the test instances except P12 under 5 min CT have resulted with sole Pareto solution. However, the nature of Pareto solution (sole or multiple) depends on the data.
- The Pareto front with two solutions in its front to P12 problem under 5 min CT validates the update mechanism adopted in the proposed algorithms.

## 6. Conclusions

This paper addresses a multiobjective optimization of two-sided assembly line balancing problem associated with task directions assignment restrictions for the objective criterion of minimizing the unbalance among work stations and the number of workstations. The model has the capability to address all three-task direction restrictions. When all the tasks are strictly restricted to any one side (i.e., left or right), it turns out to be a single side assembly line problem. When the tasks are restricted to either left or right, then the model becomes a two-sided assembly line problem with strict side restrictions. Hence, the model presented in this paper is a general case of an assembly line balancing problem and can be used for all types (single-sided as well two-sided) of assembly line balancing problems. Though the model presented in this paper deals with two objectives of minimum number of workstations and minimum unbalance time among the workstations, it may be extended to include objectives. Two algorithms, namely, EHA and SAA, are proposed to solve the problem for the two objectives of minimum number of workstations and minimum unbalance time among the workstations. Both algorithms use the logic of Largest Candidate Ranking Algorithm for assignment of tasks in the workstations. Other heuristics may provide better solution quality and may be attempted. The proposed algorithms are structured such that they can be used for any other two objectives by changing the evaluation parameters suitably to go with the selected objectives. The algorithm structured based on EHA provides reasonable quality assignment of tasks to workstations to small size problems in practical time and can be useful in dynamic environments. However, the computational effort is very high for large size practical problems. On the other hand, the proposed SAA has the capability to explore the large solution space with limited number of searches to locate near optimal solution. Though the results of SAA show that SAA is better for larger size problems, its robustness and computational efficiency can be improved by fine tuning the parameters. The future work may consider fine tuning of SAA by applying different perturbation mechanisms and SA parameters. Besides, other metaheuristics, as alternate to SAA, may be attempted for large problem instances and more than two objectives.

## Figures and Tables

**Table 1:** Input data of P19 TALBP.

Task “*i*”	Processing time “*t*_*i*_” (min)	Task direction “*k*_*i*_”	Code of task directions	Number of precedence “nop_*i*_”	List of immediate precedence “*p*_*i*_”
1	2.8	L	1	0	—
2	3.1	R	2	0	—
3	2.5	E	3	1	1
4	3.4	R	2	2	1, 2
5	3.2	L	1	1	3
6	2.7	E	3	1	4
7	2.6	L	1	1	4
8	3.3	L	1	1	4
9	5.9	E	3	2	5, 6
10	3.7	R	2	2	6, 7
11	4.1	L	1	2	7, 8
12	2.2	E	3	2	9, 10
13	1.8	R	2	1	10
14	1.2	R	2	2	10, 11
15	2.3	E	3	1	12
16	2.4	L	1	2	12, 13
17	5.8	R	2	1	14
18	3.8	E	3	3	15, 16, 17
19	2.1	L	1	1	18

**Table 2:** Possible assignment of “E” type task to P19 problem.

Assignment number	“E” type tasks					
	3	6	9	12	15	18
1	L	L	L	L	L	L
2	R	L	L	L	L	L
3	L	R	L	L	L	L
4	R	R	L	L	L	L
5	L	L	R	L	L	L
⋮	⋮	⋮	⋮	⋮	⋮	⋮
17	L	L	L	L	R	L
⋮	⋮	⋮	⋮	⋮	⋮	⋮
21	L	L	R	L	R	L
⋮	⋮	⋮	⋮	⋮	⋮	⋮
64	R	R	R	R	R	R

**Table 3:** Allocation of tasks for Ist assignment of P19 (CT = 6 min).

W/S type	Allotted tasks	Processing time for task “*i*”	Work load at each station	Idle time at each station
W/S(L)-1	1	2.80	2.80	3.20
W/S(R)-1	2	3.10	3.10	2.90
W/S(L)-2	—	—	—	—
W/S(R)-2	4	3.40	3.40	2.60
W/S(L)-3	8 6	3.30 2.70	6.00	0.00
W/S(R)-3	—	—	—	—
W/S(L)-4	7	2.60	2.60	3.40
W/S(R)-4	—	—	—	—
W/S(L)-5	11	4.10	4.10	1.90
W/S(R)-5	10 13	3.70 1.80	5.50	0.50
W/S(L)-6	3 5	2.50 3.20	5.70	0.30
W/S(R)-6	14	1.20	1.20	**4.80**
W/S(L)-7	9	5.90	5.90	0.10
W/S(R)-7	17	5.80	5.80	0.20
W/S(L)-8	12 16	2.20 2.40	4.60	1.40
W/S(R)-8	—	—	—	—
W/S(L)-9	15	2.30	2.30	3.70
W/S(R)-9	—	—	—	—
W/S(L)-10	18 19	3.80 2.10	5.90	0.10
W/S(R)-10	—	—	—	—

**Table 4:** Allocation of tasks for 2nd assignment of P19 (CT = 6 min).

W/S type	Allotted tasks	Processing time for task “*i*”	Work load at each station	Idle time at each station
W/S(L)-1	1 3	2.80 2.50	5.30	0.70
W/S(R)-1	2	3.10	3.10	2.90
W/S(L)-2	—	—	—	—
W/S(R)-2	4	3.40	3.40	2.60
W/S(L)-3	8 7	3.30 2.60	5.90	0.10
W/S(R)-3	6	2.70	2.70	3.30
W/S(L)-4	11	4.10	4.10	1.90
W/S(R)-4	10 13	3.70 1.80	5.50	0.50
W/S(L)-5	5	3.20	3.20	2.80
W/S(R)-5	14	1.20	1.20	4.80
W/S(L)-6	9	5.90	5.90	0.10
W/S(R)-6	17	5.80	5.80	0.20
W/S(L)-7	12 16	2.20 2.40	4.60	1.40
W/S(R)-7	—	—	—	—
W/S(L)-8	15	2.30	2.30	3.70
W/S(R)-8	—	—	—	—
W/S(L)-9	18 19	3.80 2.10	5.90	0.10
W/S(R)-9	—	—	—	—

**Table 5:** Allocation of tasks for 3rd assignment of P19 (CT = 6 min).

W/S type	Allotted tasks	Processing time for task “*i*”	Work load at each station	Idle time at each station
W/S(L)-1	1 3	2.80 2.50	5.30	0.70
W/S(R)-1	2	3.10	3.10	2.90
W/S(L)-2	—	—	—	—
W/S(R)-2	4	3.40	3.40	2.60
W/S(L)-3	8 6	3.30 2.70	6.00	0.00
W/S(R)-3	—	—	—	—
W/S(L)-4	5 7	3.20 2.60	5.80	0.20
W/S(R)-4	—	—	—	—
W/S(L)-5	11	4.10	4.10	1.90
W/S(R)-5	9	5.90	5.90	0.10
W/S(L)-6	—	—	—	—
W/S(R)-6	10 13	3.70 1.80	5.50	0.50
W/S(L)-7	12 16	2.20 2.40	4.60	1.40
W/S(R)-7	14	1.20	1.20	4.80
W/S(L)-8	15	2.30	2.30	3.70
W/S(R)-8	17	5.80	5.80	0.20
W/S(L)-9	18 19	3.80 2.10	5.90	0.10
W/S(R)-9	—	—	—	—

**Table 6:** Allocation of tasks for 9th assignment of P19 (CT = 6 min).

W/S type	Allotted tasks	Processing time for task “*i*”	Work load at each station	Idle time at each station
W/S(L)-1	1 3	2.80 2.50	5.30	0.70
W/S(R)-1	2	3.10	3.10	2.90
W/S(L)-2	—	—	—	—
W/S(R)-2	4	3.40	3.40	2.60
W/S(L)-3	8 6	3.30 2.70	6.00	0.00
W/S(R)-3	—	—	—	—
W/S(L)-4	5 7	3.20 2.60	5.80	0.20
W/S(R)-4	—	—	—	—
W/S(L)-5	9	5.90	5.90	0.10
W/S(R)-5	10 13	3.70 1.80	5.50	0.50
W/S(L)-6	11	4.10	4.10	1.90
W/S(R)-6	—	—	—	—
W/S(L)-7	12 16	2.20 2.40	4.60	1.40
W/S(R)-7	15 14	2.30 1.20	3.50	2.50
W/S(L)-8	—	—	—	—
W/S(R)-8	17	5.80	5.80	0.20
W/S(L)-9	18 19	3.80 2.10	5.90	0.10
W/S(R)-9	—	—	—	—

**Table 7:** Allocation of tasks for 11th sssignment of P19 (CT = 6 min).

W/S type	Allotted tasks	Processing time for task “*i*”	Work load at each station	Idle time at each station
W/S(L)-1	1 3	2.80 2.50	5.30	0.70
W/S(R)-1	2	3.10	3.10	2.90
W/S(L)-2	—	—	—	—
W/S(R)-2	4	3.40	3.40	2.60
W/S(L)-3	8 6	3.30 2.70	6.00	0.00
W/S(R)-3	—	—	—	—
W/S(L)-4	5 7	3.20 2.60	5.80	0.20
W/S(R)-4	—	—	—	—
W/S(L)-5	11	4.10	4.10	1.90
W/S(R)-5	9	5.90	5.90	0.10
W/S(L)-6	—	—	—	—
W/S(R)-6	10 13	3.70 1.80	5.50	0.50
W/S(L)-7	12 16	2.20 2.40	4.60	1.40
W/S(R)-7	15 14	2.30 1.20	3.50	2.50
W/S(L)-8	—	—	—	—
W/S(R)-8	17	5.80	5.80	0.20
W/S(L)-9	18 19	3.80 2.10	5.90	0.10
W/S(R)-9	—	—	—	—

**Table 8:** Input data for TALBP P47 shown in Figure 7 (CT = 102 seconds).

Task “*i*”	Processing time “*t*_*i*_” (seconds)	Code of task directions “*k*_*i*_”	Number of precedence “nop_*i*_”	List of immediate precedence “*p*_*i*_”
1	5.89	3	0	—
2	5.81	3	1	6
3	15.47	2	1	6
4	15.24	3	1	6
5	13.21	3	1	1
6	8.55	3	1	1
7	35.19	1	1	6
8	9.54	1	2	5, 6
9	6.33	1	1	6
10	10.12	3	1	12
11	7.53	2	1	6
12	10.40	2	1	16
13	12.29	2	1	16
14	8.77	2	1	16
15	9.91	1	1	6
16	33.14	3	1	15
17	34.93	2	2	7, 9
18	28.74	2	1	17
19	23.14	2	1	6
20	38.35	3	1	17
21	14.70	1	1	17
22	5.84	1	1	17
23	26.98	2	1	28
24	12.78	2	1	23
25	7.99	2	1	24
26	10.26	2	1	25
27	6.35	1	1	6
28	3.31	1	2	20, 21
29	5.39	1	1	23
30	19.12	1	1	28
31	8.85	1	2	23, 27
32	6.58	1	1	31
33	29.18	3	1	34
34	46.20	3	0	—
35	17.96	3	1	34
36	35.37	3	2	33, 35
37	28.41	1	1	7
38	10.60	1	1	37
39	6.19	1	1	6
40	24.96	3	1	7
41	10.54	3	1	40
42	29.70	3	0	—
43	9.68	3	2	7, 36
44	21.03	3	2	36, 42
45	14.39	3	1	44
46	8.66	3	1	45
47	14.11	3	1	44

**Table 9:** Initial seed “*X*” of the illustration problem P47.

Title	Initial feasible string for 20 “E” type tasks “*X*” which are randomly assigned to left or right side																			
Task “*i*”	**1**	**2**	**4**	**5**	**6**	**10**	**16**	**20**	**33**	**34**	**35**	**36**	**40**	**41**	**42**	**43**	**44**	**45**	**46**	**47**
Position “*j*”	1	2	3	4	5	6	7	8	9	10	11	12	13	14	15	16	17	18	19	20
*k*_*i*_(*X*)	1	1	1	1	1	1	2	2	2	1	1	1	1	1	2	2	2	2	2	1

**Table 10:** Perturbed seed “*X*_*p*_”.

Task “*i*”	**1**	**2**	**4**	**5**	**6**	**10**	**16**	**20**	**33**	**34**	**35**	**36**	**40**	**41**	**42**	**43**	**44**	**45**	**46**	**47**
Position “*j*”	1	2	3	**4**	5	6	**7**	8	9	10	**11**	12	13	14	15	**16**	17	18	**19**	20
*K*_*i*_(*X*)	1	1	1	**1**	1	1	2	2	2	1	**1**	1	1	1	2	**2**	2	2	**2**	1
*K*_*i*_(*X*_*p*_)	1	1	1	**2**	1	2	2	1	2	1	**2**	1	1	1	2	**1**	2	1	**1**	1

**Table 11:** Results of EHA, SAA, and source paper for TALBP.

Problem identifier	CT min	Minimum number of workstations							Minimum unbalance time										
		Source Paper	EHA	SAA						SAA (*n* ^2^ as perturbation termination)					SAA (*n* ^3^ as perturbation termination)				
				Trail number					EHA	Trail number					Trail number				
				1	2	3	4	5		1	2	3	4	5	1	2	3	4	5
P9 (Kim et al., 2009 [2])	**4**	5	5	5	5	5	5	5	0.99	0.99	0.99	0.99	0.99	0.99	0.99	0.99	0.99	0.99	0.99
	**5**	4	4	4	4	4	4	4	0.99	0.99	0.99	0.99	0.99	0.99	0.99	0.99	0.99	0.99	0.99
	**6**	3	3	3	3	3	3	3	0.97	0.97	0.97	0.97	0.97	0.97	0.97	0.97	0.97	0.97	0.97

P12 (Kim et al., 2009 [2])	**5**	6	6 7	6 7	6 7	6 7	6 7	6 7	2.00 1.05	2.00 1.05	2.00 1.05	2.00 1.05	2.00 1.05	2.00 1.05	2.00 1.05	2.00 1.05	2.00 1.05	2.00 1.05	2.00 1.05
	**6**	5	5	5	5	5	5	5	1.96	1.96	1.96	1.96	1.96	1.96	1.96	1.96	1.96	1.96	1.96
	**7**	4	4	4	4	4	4	4	3.01	3.01	3.01	3.01	3.01	3.01	3.01	3.01	3.01	3.01	3.01

P19	**6**	—	12	12	12	12	12	12	2.90	2.90	2.90	2.90	2.90	2.90	2.90	2.90	2.90	2.90	2.90

P24 (Kim et al., 2009 [2])	**20**	8	8	8	8	8	8	8	0.99	1.98	0.99	1.98	0.99	0.99	0.99	0.99	0.99	0.99	0.99
	**25**	6	7	7	7	7	7	7	6.00	6.00	7.00	7.02	6.00	7.00	6.00	6.00	6.00	6.00	6.00
	**30**	5	6	6	6	6	6	6	9.06	9.06	9.06	9.06	10.99	9.06	9.06	9.06	9.06	10.99	9.06
	**35**	5	5	5	5	5	5	5	12.98	15.98	12.98	16.48	15.98	12.98	15.98	12.98	12.98	15.98	12.98
	**40**	4	4	4	4	4	4	4	7.00	7.00	7.00	12.95	12.95	7.00	7.00	7.00	7.00	7.00	7.00

P47 [14]	**102/60**	8	—	8	8	8	8	8	—	18.02	19.68	16.74	14.69	18.29	14.69	14.69	16.74	14.69	14.69

**Table 12:** Data of problem P9, CT = 4, 5, and 6 min [2].

Task “*i*”	Processing time “*t*_*i*_” (min)	Code of task directions “*k*_*i*_”	Number of precedence “nop_*i*_”	List of immediate precedence “*p*_*i*_”
1	2.00	1	0	—
2	3.00	2	0	—
3	2.00	3	0	—
4	3.00	1	1	1
5	1.00	2	1	2
6	1.00	3	2	2 3
7	2.00	3	2	4 5
8	2.00	1	1	5
9	1.00	3	1	6

**Table 13:** Data of P12, CT = 5, 6, and 7 min [2].

Task “*i*”	Processing time “*t*_*i*_” (min)	Code of task directions “*k*_*i*_”	Number of precedence “nop_*i*_”	List of immediate precedence “*p*_*i*_”
1	2.00	1	0	—
2	3.00	2	0	—
3	2.00	3	0	—
4	3.00	1	1	1
5	1.00	3	1	2
6	1.00	1	1	3
7	3.00	3	2	4 5
8	3.00	2	1	5
9	2.00	3	2	5 6
10	2.00	3	2	7 8
11	2.00	3	1	9
12	1.00	2	1	11

**Table 14:** Data of P24, CT = 20, 25, 30, 35, and 40 min [2].

Task “*i*”	Processing time “*t*_*i*_” (min)	Code of task directions “*k*_*i*_”	Number of precedence “nop_*i*_”	List of immediate precedence “*p*_*i*_”
1	3.00	1	0	—
2	7.00	1	0	—
3	7.00	2	0	—
4	5.00	2	0	—
5	4.00	1	1	2
6	3.00	3	2	2 3
7	4.00	2	1	3
8	3.00	3	1	5
9	6.00	3	1	6
10	4.00	3	1	7
11	4.00	1	1	1
12	3.00	1	2	8 9
13	3.00	3	1	9
14	9.00	2	2	9 10
15	5.00	2	1	4
16	9.00	1	1	11
17	2.00	3	1	12
18	7.00	3	1	13
19	9.00	3	2	13 14
20	9.00	2	1	15
21	8.00	1	2	16 17
22	8.00	3	1	18
23	9.00	2	2	19 20
24	9.00	3	1	20

## Nomenclature

*C*: Counter
CT: Cycle time
E: Set of tasks which can be performed at either side of a station
Δ*E*: Change in entropy
I: Identifier for task
IT: Iterations number
*k*_*i*_: Task direction for *i*th task {*k*_*i*_ = L(1), R(2), E(3)}
L: Set of tasks which should be performed at a left-side station
*N*: Number of tasks
*n*: Number of “E” type task
nop_*i*_: Number of immediate predecessor of task “*i*"
*P*: Probability of acceptance
*p*_*i*_: List of predecessor for task “*i*"
R: Set of tasks which should be performed at a right-side station
*r*: Random number
*T*_*i*_: Initial temperature
*T*_*f*_: Final temperature
*T*_ek_: Individual work element task times
*t*_*i*_: Processing time of task “*i*"
UB: Unbalance time among workstations
W/S: Number of workstations
*X*: Current solution
*X*_*g*_: Pareto solution
*X*_*p*_: Perturbation solution
*Z*: Quench rate.

## References

[1] Lee TO, Kim Y, Kim YK (2001). Two-sided assembly line balancing to maximize work relatedness and slackness. *Computers and Industrial Engineering*.

[2] Kim YK, Song WS, Kim JH (2009). A mathematical model and a genetic algorithm for two-sided assembly line balancing. *Computers and Operations Research*.

[3] Bartholdi JJ (1993). Balancing two-sided assembly lines: a case study. *International Journal of Production Research*.

[4] Simaria AS, Vilarinho PM (2009). 2-ANTBAL: an ant colony optimisation algorithm for balancing two-sided assembly lines. *Computers and Industrial Engineering*.

[5] Kim YK, Kim Y, Kim YJ (2000). Two-sided assembly line balancing: a genetic algorithm approach. *Production Planning and Control*.

[6] Baykasoglu A, Dereli T (2008). Two-sided assembly line balancing using an ant-colony-based heuristic. *International Journal of Advanced Manufacturing Technology*.

[7] Simaria AS, Vilarinho PM (2004). A genetic algorithm based approach to the mixed-model assembly line balancing problem of type II. *Computers and Industrial Engineering*.

[8] Özcan U, Toklu B (2009). Multiple-criteria decision-making in two-sided assembly line balancing: a goal programming and a fuzzy goal programming models. *Computers and Operations Research*.

[9] Özcan U (2010). Balancing stochastic two-sided assembly lines: a chance-constrained, piecewise-linear, mixed integer program and a simulated annealing algorithm. *European Journal of Operational Research*.

[10] Xiaofeng H, Erfei W, Jinsong B, Ye J (2010). A branch-and-bound algorithm to minimize the line length of a two-sided assembly line. *European Journal of Operational Research*.

[11] Hu X, Wu E, Jin Y (2008). A station-oriented enumerative algorithm for two-sided assembly line balancing. *European Journal of Operational Research*.

[12] Konak A, Coit DW, Smith AE (2006). Multi-objective optimization using genetic algorithms: a tutorial. *Reliability Engineering and System Safety*.

[13] Fleszar K, Hindi KS (2003). An enumerative heuristic and reduction methods for the assembly line balancing problem. *European Journal of Operational Research*.

[14] Rubiano-Ovalle Ó, Arroyo-Almanza A (2009). Solving a two-sided assembly line balancing problem using memetic algorithms. *Ingeniería y Universidad*.

[15] Purnomo HD, Wee H-M, Rau H (2013). Two-sided assembly lines balancing with assignment restrictions. *Mathematical and Computer Modelling*.

[16] Groover MP (2002). *Automation Production Systems and Computer Integrated Manufacturing*.

[17] Van Laarhoven PJM, Aarts EHL (1987). *Annealing: Theory and Applications*.

[18] Kirkpatrick S, Gelatt CD, Vecchi MP (1983). Optimization by simulated annealing. *Science*.

[19] Fleischer M Simulated annealing: past, present, and future.

[20] Kanagaraj G, Jawahar N (2011). Optimal redundancy allocation for a reliability-based total cost of ownership model using genetic algorithm. *International Journal of Reliability and Safety*.

